# Enhancing the high-cycle fatigue performance of GH4169 alloy by eliminating structural stress concentration through high-energy impact composite modification

**DOI:** 10.1016/j.fmre.2025.02.006

**Published:** 2025-05-30

**Authors:** Qiang Wang, Guoxin Lu, Ayder Nabiev, Oday I. Abdullah, Zhong Ji, Zhong Chen

**Affiliations:** a Aviation Key Laboratory of Science and Technology on Advanced Corrosion and Protection for Aviation Material, AECC Beijing Institute of Aeronautical Materials, Beijing 100095, China; b MOE Key Laboratory for Liquid-Solid Structural Evolution and Processing of Materials, Shandong University, Jinan 250061, China; c Institute of Mechanics and Seismic Stability of Structures of the Academy of Sciences of the Republic of Uzbekistan, Department of Theory of Mechanisms and Machines, Tashkent 100125, Uzbekistan; d College of Engineering, Al-Naji University, Baghdad 10015, Iraq; e Department of Energy Engineering, College of Engineering, University of Baghdad, Baghdad 10071, Iraq; f Department of Mechanics, Al-Farabi Kazakh National University, Almaty 050040, Kazakhstan; g Jiangsu Key Laboratory of Advanced Manufacturing Technology, Huaiyin Institute of Technology, Huai’an 223001, China

**Keywords:** High-energy modification, Laser shock peening, GH4169 superalloy, Residual stress, High-cycle fatigue, S-N curve

## Abstract

Despite the advancements of traditional surface treatments like shot peening (SP) and laser shock peening (LSP) in enhancing the fatigue life of GH4169 superalloy, these techniques remain limited, particularly under high-stress concentration conditions. This study introduces a novel high-energy impact composite modification method that integrates SP and LSP to comprehensively address the fatigue strength sensitivity to stress concentration, a persistent issue for GH4169 in high-cycle fatigue applications. For the modified specimens, the fatigue limit under high-stress concentration conditions of *Kt* = 3 increased from 215 MPa to 517 MPa, more than doubling. Compared to grinding, composite modification shifts fatigue crack initiation from the surface to subsurface regions, demonstrating superior fatigue resistance. The high-intensity surface modification eliminates structural stress concentration as the primary factor determining the fatigue life of the material. The concentrated stress at the carbide phase interfaces within the material, which cannot be sufficiently weakened by residual compressive stress, becomes the new weak point. The study concludes that high-energy impact composite modification offers a viable method to enhance the durability and performance of GH4169 superalloys in demanding applications.

## Graphical abstract

**Figure:**
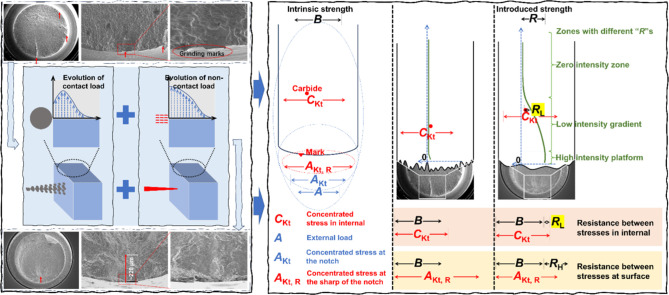

## 1 Introduction

GH4169 superalloy is extensively used in aerospace and high-performance engineering applications due to its exceptional mechanical properties and high-temperature performance [[1], [2], [3]]. However, a significant limitation of GH4169 lies in its sensitivity to stress concentration during high-cycle fatigue, where localized stress can lead to crack initiation and premature component failure. Addressing this limitation is critical to ensuring the reliability and durability of components in demanding environments.

Traditional surface treatment methods, such as mechanical shot peening (SP) [4,5] and laser shock peening (LSP) [[6], [7], [8]], have been employed to improve fatigue life and reduce stress concentration sensitivity in GH4169. However, these methods have shown limited effectiveness under high-stress concentration conditions, where their ability to uniformly enhance surface integrity and mitigate stress sensitivity is constrained. For instance, Wang et al. [9] investigated the effects of aging temperature on the fatigue properties of shot-peened single-crystal superalloys, while Rodríguez et al. [10] explored the mechanisms by which LSP regulates fatigue crack growth in Inconel 718. Despite these advances, neither approach fully addressed stress concentration effects or consistently shifted fatigue crack initiation away from critical surface regions.

Recent studies underscore the persistent challenge of addressing stress concentration sensitivity in high-cycle fatigue applications of nickel-based alloys, particularly under complex loading conditions. Surface modifications such *As sp* and LSP primarily target residual stress enhancement and surface hardening but often fail to address intrinsic microstructural stress concentration at critical phase interfaces. This limitation calls for innovative solutions capable of providing more comprehensive enhancements to surface integrity and fatigue resistance.

This study introduces a novel high-energy impact composite modification method that combines SP and LSP to enhance the fatigue resistance of GH4169 superalloy. This innovative approach aims to combine the benefits of SP and LSP, leading to improvements in surface integrity, residual stress distribution, and fatigue behavior. By examining the changes in surface topography, micromechanical properties, and fatigue performance, this research seeks to provide a comprehensive understanding of the benefits of this advanced surface modification technique.

## 2 Materials and methods

### 2.1 Preparation of experimental materials

#### 2.1.1 Test materials and specimen preparation

Nickel-based superalloy GH4169 was chosen as the experimental material. The composition, heat treatment process [11], and related mechanical properties [12] of this material can be found in several published papers [11,12]. The specimens for surface integrity analysis had dimensions of 10 mm × 10 mm × 8 mm. The preparation of notched rotating-bending fatigue specimens involved rough turning, fine turning, and thread grinding methods [9].

The initial state of the specimens for comparison was grinding. The specific process parameters for SP and LSP in the high-energy impact composite modification were as follows: SP treatment was conducted using a pneumatic CNC shot peening machine with an Almen intensity of 0.25 mmA, achieving 200% surface coverage. The subsequent LSP treatment utilized a 2 mm diameter circular laser spot with 6 J energy, a 10 ns pulse width, and a 50% overlap ratio both horizontally and longitudinally. The same process parameters for SP and LSP were applied to other specimens used in comparative tests, whether they underwent individual SP or LSP.

#### 2.1.2 Rotating-bending fatigue testing

A rotating-bending fatigue testing machine to perform a fatigue life test at 10^7^ cycles on notched (Stress concentration factor *Kt* = 1, 1.7, 3) fatigue specimens at room temperature, with a stress ratio *R* = −1 (*R* = *σ*_min_/*σ*_max_) and a rotating speed of 3500 RPM. Where *σ*_max_ and *σ*_min_ are the maximum and the minimum stresses during the high-cycle fatigue loading, respectively. Besides the S-N curve (Stress-life curve: S—Stress, N—Number of cycles), fracture surface analysis of the specimens should also be conducted. Therefore, additional fatigue tests at higher stress levels are also carried out. To expedite obtaining fracture specimens, specimens with different modification states are subjected to different maximum stresses (*σ*_max_) in fatigue testing.

### 2.2 Experimental characterization and analysis

#### 2.2.1 Quantitative characterization of surface topography

Surface roughness and morphology were used to characterize the surface integrity of specimens in different states. The surface roughness and morphology of a specimen were measured by the use of an optical interference profiler (Newview9000, Zygo, USA).

#### 2.2.2 Microstructural and fracture analysis

Based on the high-cycle fatigue tests, to scientifically figure out the stress concentrations resulting from fatigue cracks, the fatigue fracture after etch was examined via JEOL JSM 7610F scanning electron microscope (SEM).

#### 2.2.3 Micromechanical property testing

Micro-Vickers hardness (HV) testing was performed using a micro-hardness tester (HV-1000) to demonstrate microhardness, with three repeat measurements taken to calculate the average hardness value.

Residual stress measurements were carried out using X-ray diffraction (XRD) with the sin²ψ technique. The X-ray beam, utilizing MnKα radiation, had a diameter of approximately 1 mm, and measurements were taken on the (311) nickel diffraction peak [13] at a diffraction angle of around 152° A Poisson’s ratio of 0.31 was used for stress calculation, and the feed angle for ladder scanning was set at 0.1°/s.

## 3 Results

### 3.1 Changes in surface integrity

**Fig. 1:**
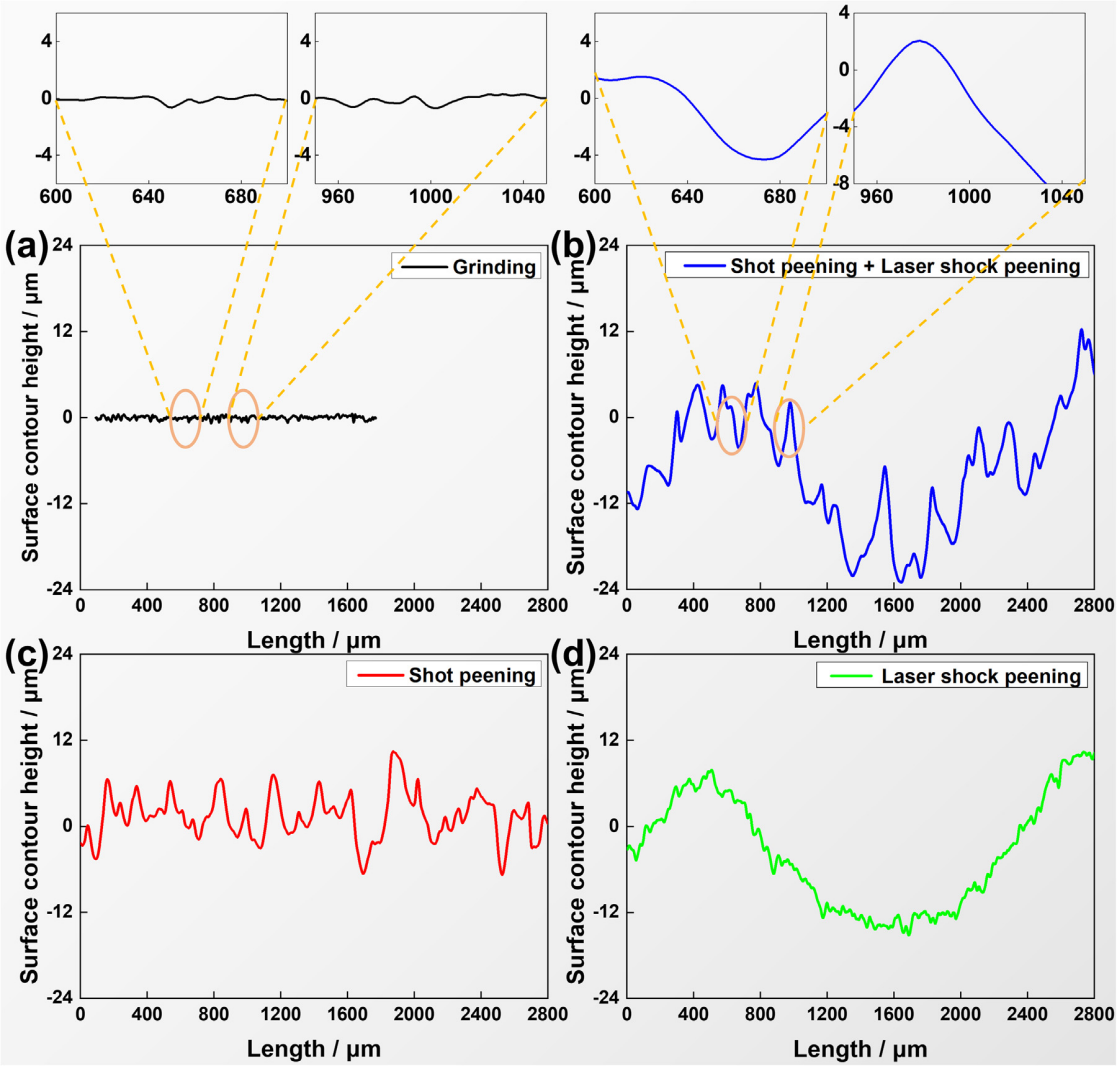
**Surface morphologies induced by different post-treatment methods**. (a) Grinding: parallel machining marks visible; (b) High-energy composite modification: large-scale undulations with smooth transitions; (c) Single SP: increased surface roughness; (d) Single LSP: formation of macroscopically visible craters.

Fig. 1 was constructed to illustrate the changes in surface morphology caused by different post-treatment states. Additionally, a set of specimens was specifically used for the analysis and testing of relevant indicators on the outermost surface. The corresponding data are listed in Table 1. Similarly, to ensure the accuracy of the analysis, the changes in surface integrity-related indicators induced by individual SP and LSP were also obtained simultaneously for comparative discussion.

**Table 1:** **Comparison of micromechanical properties after different post-treatment processes**.

Surface treatment methods	Microhardness / HV	Residual stress / MPa	
		X-direction	Y-direction
Grinding	461	−219	−107
Shot peening	500	−955	−923
Laser shock peening	519	−1455	−1459
Shot peening +			
Laser shock peening	523	−1425	−1405

Residual stresses (MPa) are measured in the X-direction (parallel to machining marks) and Y-direction (perpendicular to machining marks). Data include microhardness (HV) values.

The grinding surface morphology consists of fine, parallel machining marks, with an average surface hardness of HV 461. The average surface residual stress value in the direction parallel to the machining marks is −219 MPa, while in the perpendicular direction, it is −107 MPa. SP eliminates the grinding marks, but the impact of the projectiles significantly increases surface roughness. The average surface hardness is HV 500, which is 39 HV higher than grinding, and the surface residual stress exceeds −900 MPa, increasing by over 700 MPa compared to grinding. LSP does not visibly eliminate the grinding marks; instead, macroscopically visible impact craters form on the surface. The average surface hardness is 519 HV, and the average surface residual stress exceeds −1,450 MPa. High-energy composite-modification surface morphology integrates features of both SP and LSP. It conceals the grinding marks while forming large-scale undulations. At the same time, it induces microhardness and residual compressive stress levels comparable to those of LSP.

### 3.2 Fracture characteristics of fatigue failure

**Fig. 2:**
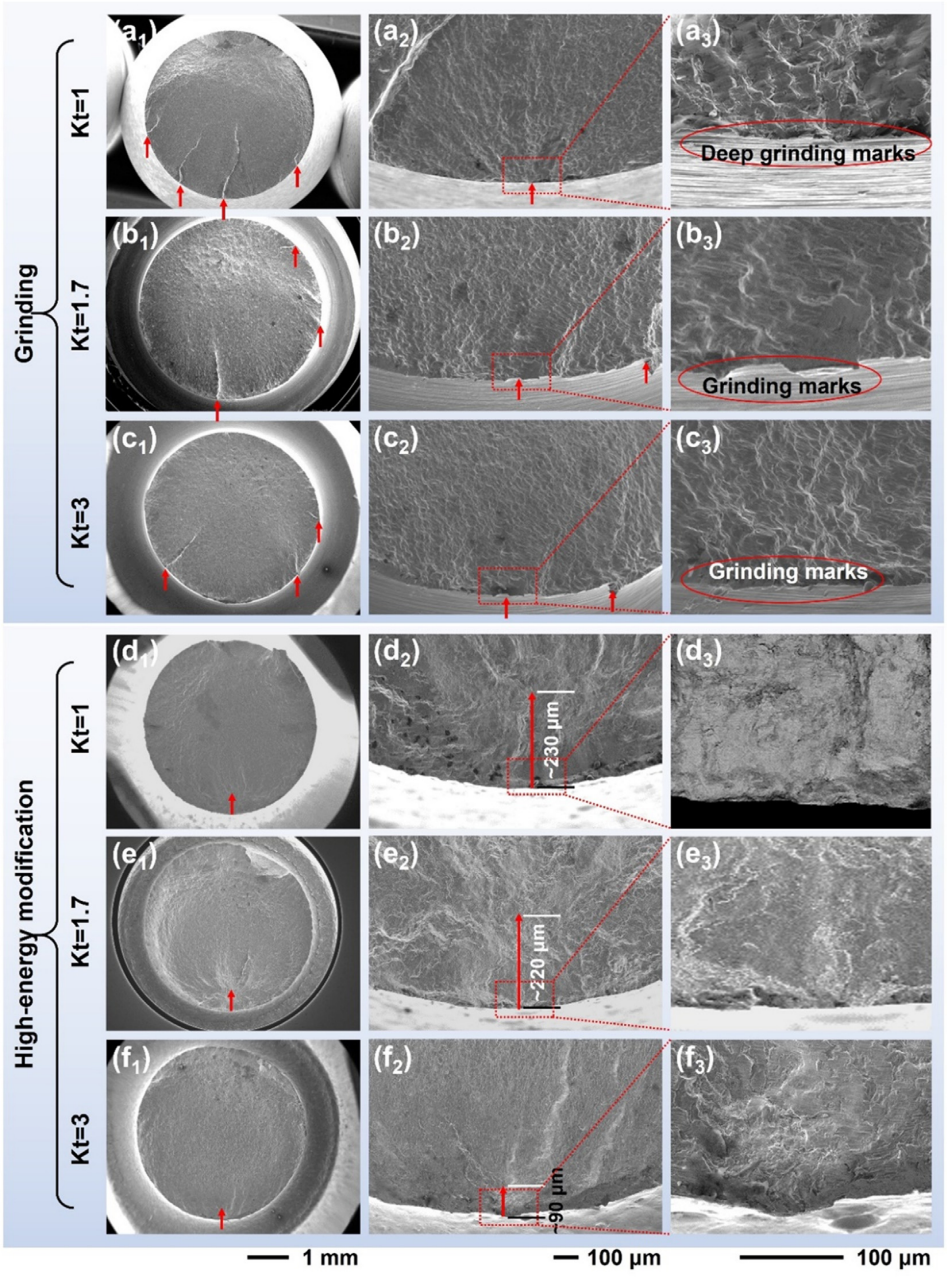
**Fracture morphologies of specimens with different *Kt* values under high-cycle fatigue**. (a1, b1, …, f1): Overall fracture contours; (a2, b2, …, f2): Crack initiation sites; (a3, b3, …, f3): Magnified morphology of crack origins.

Considering the issue of the specimens undergoing continuous loading during high-cycle fatigue testing, several specimens were subjected to a specific level of *σ*_max_, leading to fracture failure after a limited number of fatigue cycles. The applied stresses and the final fatigue lives of these specimens are listed in Table 2, and the fracture morphologies are shown in Fig. 2.

**Table 2:** **The corresponding test conditions used to obtain fatigue fractures of different specimens**.

No.	*σ*_max_ / MPa	Fatigue life / Number of cycles
a	750	8.65 × 10^4^
b	450	1.2 × 10^4^
c	360	18.8 × 10^4^
d	800	218 × 10^4^
e	685	198 × 10^4^
f	620	216 × 10^4^

The letters a, b, and c represent the grinding specimens under different *Kt* values, while d, e, and f correspond to the respective specimens treated with high-energy modification.

Combining the surface post-treatment conditions shown in Fig. 2 and the stress concentration characteristics of the notched specimens, the data in Table 2 still demonstrate the significant surface life-extension effect brought by high-energy composite modification. Regardless of grinding or high-energy surface modification, the larger the *Kt* value, the more significantly the fatigue life of the specimen is affected by the stress concentration effect. Therefore, when setting the *σ*_max_, the stress level applied to notched specimens with a high *Kt* value was reduced to some extent. It can be seen that, when the *Kt* values are the same, the high-energy impact modified specimens, despite being subjected to higher *σ*_max_, exhibit significantly improved fatigue life compared to the ground specimens. This conclusion is consistent with the results shown in Table 3, directly confirming the positive effect of high-energy surface modification on fatigue life extension.

**Table 3:** **Rotating-bending fatigue limits (MPa) of ground and high-energy surface-modified GH4169 specimens**.

Surface treatment methods	*Kt* = 1	*Kt* = 1.7	*Kt* = 3
Grinding	494	268	215
Shot peening + laser shock peening	-	563	517

Fig. 2 provides an overview of the fracture morphologies in different regions of the fatigue fracture specimens. These images depict the changes in fracture characteristics, highlighting differences in crack initiation due to the varying *Kt* values and the effects of high-energy composite modification. It is important to note that, based on the dimples in the instant fracture zone and the fatigue striations in the propagation zone of these fractured specimens, it can be concluded that ductile fracture occurred under high-cycle fatigue testing conditions [14,15]. Other features beyond the crack initiation are not the focus of analysis and discussion in this study.

The fracture characteristics of fatigue specimens before and after high-energy modification were comparatively analyzed. For ground specimens with *Kt* = 1, fatigue failure occurred in a surface multi-origin mode, originating from deeper machining marks on the surface. Specimens with *Kt* = 1.7 and *Kt* = 3 exhibited surface multi-origin fatigue failure, originating from discontinuities in the surface machining marks. After high-energy surface modification, the fatigue failure of specimens with different *Kt* values consistently displayed a subsurface single-origin mode, originating from certain special phases on the subsurface. For the fatigue specimens prepared with three different *Kt* values, their crack origins are all located outside the shallow high-intensity zone (away from the outermost surface of about 50µm) after high-energy modification. However, an increase in the value of *Kt* will still lead to the crack origin gradually approaching the surface.

### 3.3 High-cycle fatigue behavior

**Fig. 3:**
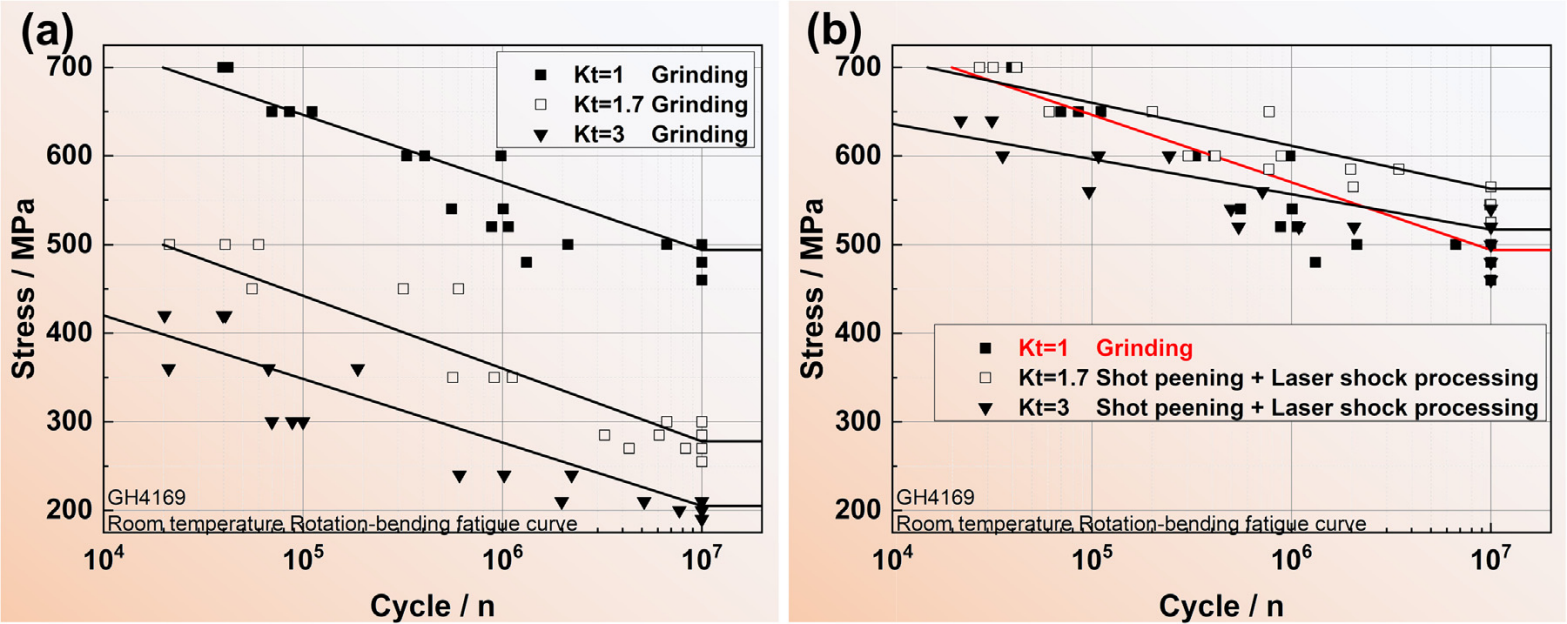
**S-N curves of ground and high-energy impact composite-modified specimens under rotating-bending fatigue conditions**. Axes: Stress (MPa) versus Number of cycles (log scale). High-energy composite modification demonstrates superior fatigue resistance across different Kt values.

Rotating-bending fatigue tests under alternating loads are an important method for characterizing the high-cycle fatigue life of superalloy materials [14,16]. To directly analyze the application effects of high-energy impact composite modification, rotating-bending fatigue experiments were conducted on GH4169 specimens before and after high-energy modification, resulting in the corresponding S-N curves (Fig. 3). It is worth noting that to address the issue of stress concentration sensitivity in superalloy materials during fatigue service, specimens with different *Kt* values were prepared. This yielded more comprehensive fatigue performance data for a stronger validation of the application effects. Based on the S-N curves in Fig. 3, the data on the room-temperature rotating-bending fatigue limit of the two methods, grinding and high-energy impact surface modification, as a function of the stress concentration factor, are listed in Table 3.

From Fig. 3 and Table 3, it can be seen that the rotating-bending fatigue limit of ground specimens with *Kt* = 1 is 494 MPa. When *Kt* = 1.7, it decreases to 268 MPa, a reduction of 45.7%. When *Kt* = 3, it further decreases to 215 MPa, a reduction of 56.5%. This demonstrates the high sensitivity of the fatigue strength of ground GH4169 specimens to stress concentration. After composite modification, the rotating-bending fatigue limit of specimens with *Kt* = 1.7 reaches 563 MPa, which is an increase of 110.1% compared to the ground specimens with *Kt* = 1.7 and a 14.0% increase compared to the ground specimens with *Kt* = 1. For specimens with *Kt* = 3, the rotating-bending fatigue limit reaches 517 MPa, an increase of 140.5% compared to the ground specimens with *Kt* = 3 and a 4.7% increase compared to the ground specimens with *Kt* = 1. The stress concentration sensitivity of room temperature fatigue strength is thus resolved.

## 4 Discussion

### 4.1 Different trends of fatigue strength variation

**Fig. 4:**
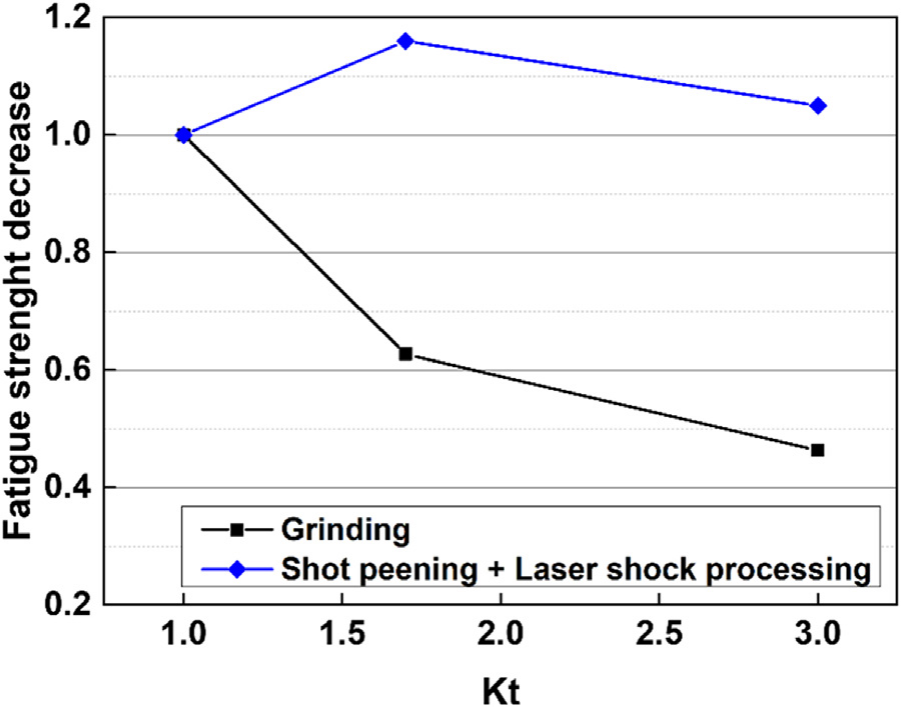
**Quantitative trend of fatigue strength attenuation with varying *Kt* values, highlighting the effectiveness of high-energy modification in maintaining high fatigue strength under increasing stress concentration**.

GH4169 alloy possesses excellent inherent comprehensive mechanical properties; however, traditional “forming” manufacturing methods cannot resolve the issue of fatigue strength sensitivity to stress concentration. For nearly a century, researchers have attempted to address this “persistent problem” using surface strengthening methods, such as shot peening, to improve surface integrity, increase fatigue life, and reduce fatigue stress concentration sensitivity. However, the effects have been very limited [17,18]. Based on Table 3, the trend of the relationship between fatigue strength variation and *Kt* value can be obtained (Fig. 4). In cases of increased *Kt* values, high-energy modification treatment prevents a decline in the fatigue strength of the specimens. The adopted high-energy composite surface modification method is an effective way to thoroughly solve the problem of fatigue strength sensitivity to stress concentration. The composite modification not only significantly enhances the fatigue strength of specimens with *Kt* = 1.7 and *Kt* = 3 but also increases the fatigue strength of high-*Kt* value specimens beyond that of ground specimens with *Kt* = 1. After composite modification, the excellent inherent mechanical properties of GH4169 material make it suitable for application under extreme conditions.

The variations in the specimens discussed are all based on the comparison with the fatigue behavior of the grinding specimens with *Kt* = 1. If the *Kt* value is simply increased, the stress concentration effect on the material would naturally become more pronounced, leading to a reduction in all fatigue limits. However, when the *Kt* value is increased and the material undergoes high-energy modification, the fatigue limit actually improves. A higher *Kt* value signifies more severe stress concentration, while high-energy modification has the effect of reducing stress concentration. The experimental results demonstrate that the fatigue limit of specimens with high *Kt* values improved after high-energy modification, indicating that this treatment weakens or eliminates the heightened stress concentration effects caused by the increased *Kt* value.

### 4.2 Changes in fracture mechanisms

The positions of fatigue failure initiation for bending fatigue of different specimens, as shown in Fig. 2 for grinding and high-energy composite modification, are listed in Table 4. It can be observed that the fatigue failure initiation positions of the specimens are significantly different before and after high-energy modification treatment. For specimens manufactured by grinding, fatigue failure initiates at the surface, with fatigue cracks originating starting from areas of high-stress concentration caused by incomplete or uneven grinding, such as deep machining marks or discontinuous machining mark locations. However, for specimens after high-energy impact modification, the fatigue crack initiation positions are no longer at the surface.

**Table 4:** **The failure mode (crack initiation position) of rotating-bending fatigue**.

Surface treatment methods	*Kt* = 1	*Kt* = 1.7	*Kt* = 3
Grinding	Deep machining tool marks	Discontinuous machining tool marks	Discontinuous machining tool marks
Shot peening +			
Laser shock peening	-	Carbides	Carbides

The incomplete or uneven nature of machining marks exhibits multiplicity and randomness, namely the cutting shape and depth of the grinding tool marks at different positions are very inconsistent, resulting in lower fatigue strength and higher uncertainty in fatigue performance for ground specimens. High-energy impact surface modification induces localized distribution of nano “effective grains” [19,20] and regions with high hardness and large residual compressive stress in the shallow surface layer of the specimen. The “effective grains” refers to the refined grains involved in the Hall-Petch theory, where the refinement of these grains leads to changes in the material’s strength and other mechanical properties. This significantly enhances fatigue resistance, leading to the evolution of fatigue failure in high-energy composite-modified specimens with different stress concentration factors into a subsurface mode.

**Fig. 5:**
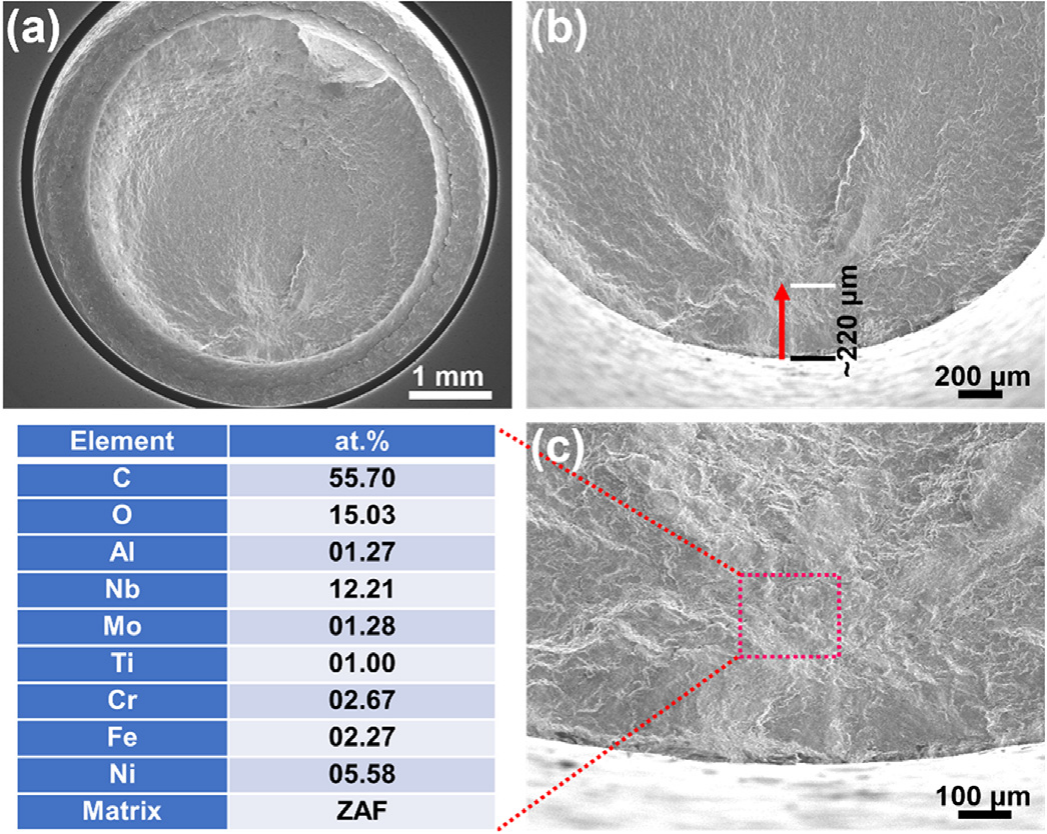
**The fracture morphology and EDS analysis results of the selected specimen treated with high-energy impact modification at *Kt* = 1.7 (specimen e in**Fig. 2**)**. (a, b): Crack initiation sites; (c): Elemental analysis showing carbide concentration at the crack origin.

For the study, a fatigue fracture specimen treated with high-energy impact modification at *Kt* = 1.7 was selected, and the crack initiation region of the specimen was subjected to energy-dispersive X-ray spectroscopy (EDS) analysis (Fig. 5). It can be observed that the C element content in this region is significantly elevated, with an atomic percentage of up to 55.70%. This indicates the presence of C-containing compounds in this region. These carbides generate additional stress concentration at the phase interfaces during fatigue, as they do not deform in conjunction with the surrounding matrix or other phases, leading to the initiation of fatigue cracks. The fact that fatigue cracks originate from carbides in the material suggests that structural stress concentration in the specimen is no longer the primary factor affecting fatigue strength. Instead, the fatigue strength of the specimen primarily depends on the intrinsic properties of the material and the performance of the surface modification layer.

Additionally, surface morphology characteristics are also considered to have a certain influence on fatigue crack initiation. When the surface morphologies of specimens a and b in Fig. 1 are locally magnified, it can be observed that grinding and high-energy composite modification result in two distinctly different types of surface morphologies. “Long-range straightness, short-range sharpness” can be used to summarize the surface morphology characteristics of the ground state material, while high-energy modification leads to a surface morphology characterized by “long-range fluctuations, short-range smoothness”. Specifically, regarding Fig. 1, the surface of the material in the entire test range shown in Fig. 1a appears flat, while the two highlighted local areas contain significantly weakened tool marks. In contrast, the overall test range in Fig. 1b is more irregular, but its local areas are relatively smooth. This indicates that while grinding causes severe local sharpness, high-energy composite modification induces relatively larger-scale undulations but with significantly lower local sharpness. This should be regarded as a positive factor for fatigue cracks not to originate at the surface of high-energy modified specimens.

**Fig. 6:**
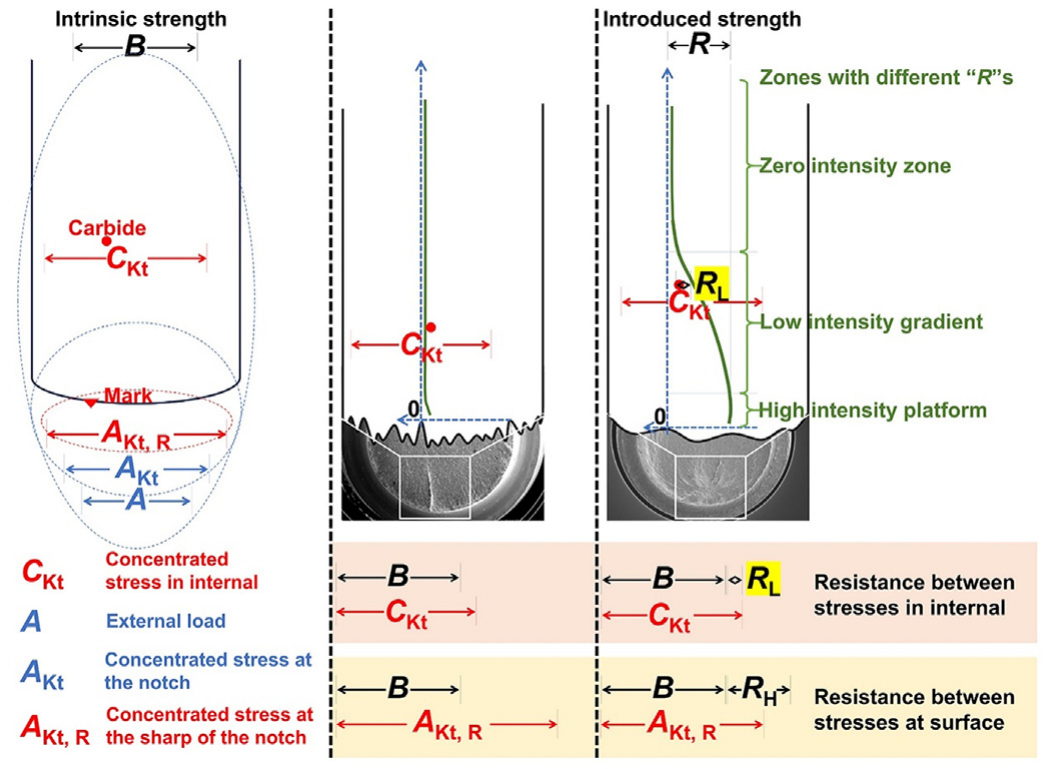
**Schematic diagram illustrating the fatigue fracture mechanism for specimens with varying surface integrity, comparing ground and high-energy modified states**.

Why does the fatigue crack origin of the specimens in the high-energy impact composite modification state shift to the interior of the material? Considering the stress concentration state on the material surface and the strengthening effect introduced by high-energy modification, Fig. 6 was constructed to explain this issue. The presence of residual stress is an important result and intrinsic manifestation of the gradient-distributed impact load and the resulting microstructural evolution. The fatigue crack origin mechanism is analyzed from the perspective of the interplay between internal and external stresses.

For the notched fatigue specimens selected in this study, when the tensile external load is set to *A*, the concentrated stress at the notch and the sharp undulation of the notch are represented by *A*_Kt_ and *A*_Kt, R_, respectively, and the concentrated stress caused by internal carbides is represented by *C*_Kt_. The actual concentrated stress loads at different locations are thus *A*_Kt, R_ and *C*_Kt_.

**Fig. 7:**
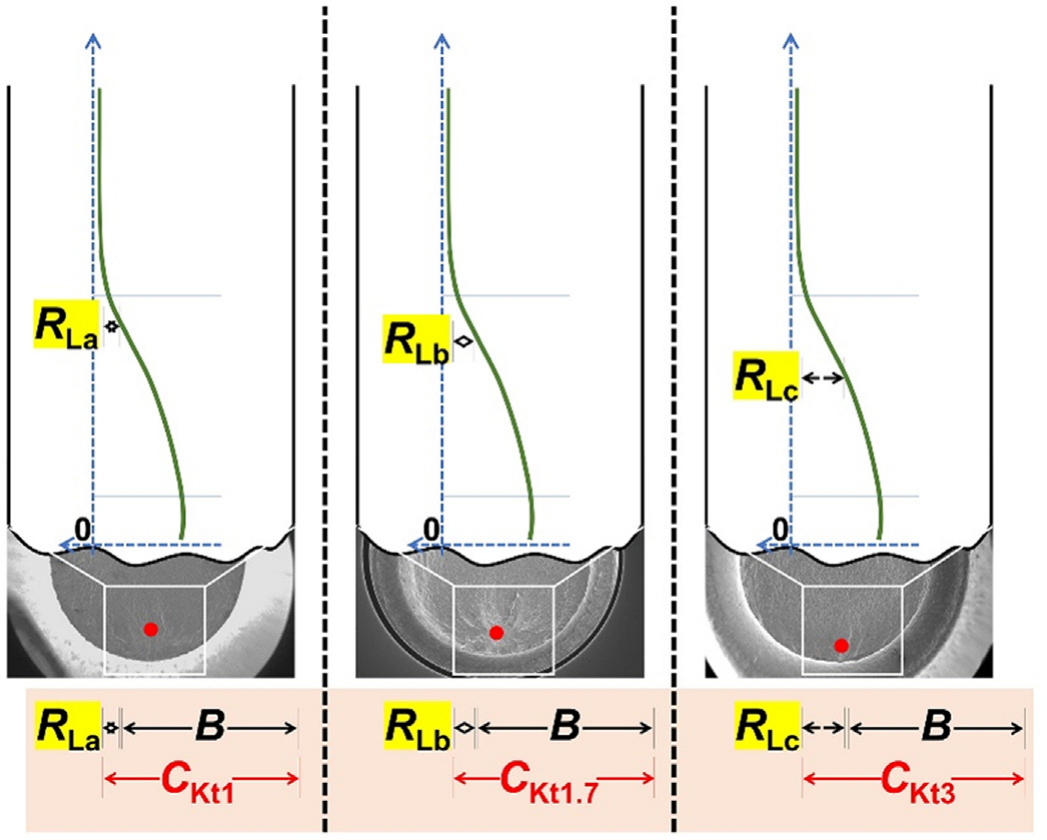
**Schematic representation of the influence of *Kt* on crack origin depth in high-energy modified specimens**. Increasing *Kt* values result in crack origins gradually shifting closer to the surface.

The inherent strength of the material and the residual strength introduced by surface modification are used to resist the concentrated stress induced by the external load. Under specific external loads, the stress from the inherent strength of the material that resists the external load is defined as *B*. The strengthening stress introduced by surface modification is represented by *R*. If the micromechanical property distribution introduced by high-energy impact composite modification (Fig. 4, Fig. 7) is divided into three regions: high-strength platform region, low-strength gradient region, and zero-strength region, the corresponding levels of *R* are defined as *R*_H_, *R*_Lx_, and 0, respectively. The mentioned three regions are as depicted as Fig. 6.

During the loading process, stress interactions occur both at the material surface and at the positions of internal carbides. The outcome of these interactions determines which area is relatively weaker. For the ground state specimens, the introduced strength *R* = 0, and only the inherent strength *B* can resist the concentrated stress caused by the external load. In this case, based on the structural stress concentration effect, the concentrated stress caused by the sharp undulation on the material surface (*A*_Kt, R_) is greater than the internal concentrated stress (*C*_Kt_). If both *A*_Kt, R_ and *C*_Kt_ are greater than *B*, then Eq. (1) holds. Based on the results and implications of Eq. (1), the fatigue crack origin must be on the material surface.

(1) $$A_{\text{Kt},\;R}-B>C_{\text{Kt}}-B$$

For specimens modified by high-energy impact surface treatment, besides *B*, the different levels of introduced strength at different locations of the material also play an important role in resisting the external load. Under the condition that *C*_Kt_ remains consistent with that of ground specimens, the sharp undulations causing *A*_Kt, R_ in high-energy modified specimens are relatively smaller due to the “short-range smoothness” surface morphology. This greatly increases the probability of Eq. (2) occurring. If the *R*_L_ at a certain internal location of the material is small, then Eq. (3) occurs, and the fatigue crack origin is located inside the material. Specifically, this position is within the deeper region beyond the high-intensity shallow layer.

(2) $$A_{\text{Kt},\;R}-\left(B+R_{H}\right)<0$$

(3) $$C_{\text{Kt}}-\left(B+R_{L}\right)>A_{\text{Kt},\;R}-\left(B+R_{H}\right)$$

Specifically, the crack origin depths shown in d2, e2, and f2 in Fig. 2 exhibit a gradual decreasing trend, indicating that the increase in *Kt* causes the crack origin to move closer to the specimen surface. Fig. 7 is used to illustrate the reason behind this phenomenon. As shown, when the *Kt* value of the notched specimen increases, the external load induces greater concentrated stress at the notch, thereby increasing the concentrated stress (*C*_Kt_) at the interfaces of internal carbides. With the inherent strength level (*B*) unchanged, larger *R*_Lx_ assistance is required to resist *C*_Kt_. The micromechanical property distribution introduced by high-energy modification under certain process parameters is consistent. When *R*_Lc_ > *R*_Lb_ > *R*_La_ in Fig. 7, their respective depths must decrease.

## 5 Conclusion

This study demonstrates the effectiveness of high-energy impact composite modification in enhancing the high-cycle fatigue performance of GH4169 superalloy. The key findings are summarized as follows:

(1) Surface Integrity Enhancement: High-energy composite modification significantly increased surface hardness and residual compressive stress compared to traditional grinding. The induced surface morphology featured large-scale undulations with minimized local sharpness, effectively reducing stress concentration effects.
(2) Fatigue Strength Improvement: The rotating-bending fatigue limit of specimens modified by the composite method showed remarkable improvements, particularly under high-stress concentration conditions (*Kt* = 1.7 and *Kt* = 3). For *Kt* = 3, the fatigue limit increased from 215 MPa to 517 MPa, more than doubling compared to ground specimens.
(3) Crack Initiation Shift: Unlike ground specimens, where fatigue cracks originated from surface machining marks, modified specimens exhibited subsurface crack initiation. This shift is attributed to the formation of a robust surface modification layer with enhanced micromechanical properties.

## Declaration of competing interest

The authors declare that they have no conflicts of interest in this work.

## References

[1] Zhenan Z., Yang W., Li L. (2023). Synchronously enhanced strength-ductility of l-DEDed GH4169 with varying energy input. Int. J. Mech. Sci..

[2] Dong Z., Ouyang P., Zhang S. (2023). Effect of building direction on anisotropy of mechanical properties of Gh4169 alloy fabricated by laser powder bed fusion. Mat. Sci. Eng. A-Struct..

[3] Qin Z., Li B., Chen C. (2023). Crack initiation mechanisms and life prediction of GH4169 superalloy in the high cycle and very high cycle fatigue regime. J. Mater. Res. Technol..

[4] Salvati E., Lunt A.J.G., Heason C.P. (2020). An analysis of fatigue failure mechanisms in an additively manufactured and shot peened IN 718 nickel superalloy. Mater. Design..

[5] Orozco-Caballero A., Jackson T., da Fonseca J.Q. (2021). High-resolution digital image correlation study of the strain localization during loading of a shot-peened RR1000 nickel-based superalloy. Acta Mater..

[6] Chin K.S., Idapalapati S., Ardi D.T. (2020). Thermal stress relaxation in shot peened and laser peened nickel-based superalloy. J. Mater. Sci. Technol..

[7] Fu W., Huang Y., Sun J. (2022). Strengthening CrFeCoNiMn_0.75_Cu_0.25_ high entropy alloy via laser shock peening. Int. J. Plast..

[8] Pan X., Zhou L., Wang C. (2023). Microstructure and residual stress modulation of 7075 aluminum alloy for improving fatigue performance by laser shock peening. Int. J. Mach. Tool. Manu..

[9] Wang X., Ma S., Hu D. (2022). Effect of aging temperature on the fatigue properties of shot-peened single crystal superalloy at intermediate temperature. Int. J. Fatigue..

[10] Rodríguez V.M., Rubio González C., García S.Flores (2023). Analysis of the effects of isothermal aging and Laser Shock peening on the flow properties, fatigue crack growth, and fracture toughness of Inconel 718. Opt. Laser Technol..

[11] Lu G., Wang Q., Attard B. (2025). Evidence of microstructural evolution linked to non-monotonic distribution of micromechanical properties induced by shot peening. J. Mater. Sci. Technol..

[12] Du J., Lü X., Deng Q. (2014). Effect of heat treatment on microstructure and mechanical properties of GH4169 superalloy. Rare Metal Mat. Eng..

[13] Kumar D., Idapalapati S., Wei W. (2019). Microstructure-mechanical property correlation in shot peened and vibro-peened Ni-based superalloy. J. Mater. Process. Technol..

[14] Kong W.-W., Yuan C., Zhang B.-N. (2020). Investigations on cyclic deformation behaviors and corresponding failure modes of a Ni-based superalloy. Mat. Sci. Eng. A-Struct..

[15] Liu G., Salvat Cantó J., Winwood S. (2018). The effects of microstructure and microtexture generated during solidification on deformation micromechanism in IN713C nickel-based superalloy. Acta Mater..

[16] Cervellon A., Hémery S., Kürnsteiner P. (2020). Crack initiation mechanisms during very high cycle fatigue of Ni-based single crystal superalloys at high temperature. Acta Mater..

[17] Qin Z., Li B., Chen R. (2023). Effect of shot peening on high cycle and very high cycle fatigue properties of Ni-based superalloys. Int. J. Fatigue..

[18] Jiang Y., Liu M., Zou T. (2024). Numerical simulation and high cycle fatigue behaviour study on shot peening of MAR-M247 nickel-based alloy. Int. J. Fatigue..

[19] Ma K., Wen H., Hu T. (2014). Mechanical behavior and strengthening mechanisms in ultrafine grain precipitation-strengthened aluminum alloy. Acta Mater..

[20] Sharma A., Song J., Furfari D. (2021). Remarkable near-surface microstructure of nanoparticles and oxide film in laser shock peened Al-Zn-Mg-Cu alloy. Scripta Mater..

